# Separating conditional and unconditional cooperation in a sequential Prisoner’s Dilemma game

**DOI:** 10.1371/journal.pone.0187952

**Published:** 2017-11-09

**Authors:** Raoul Bell, Laura Mieth, Axel Buchner

**Affiliations:** Department of Experimental Psychology, Heinrich Heine University Düsseldorf, Düsseldorf, Germany; Southwest University, CHINA

## Abstract

Most theories of social exchange distinguish between two different types of cooperation, depending on whether or not cooperation occurs conditional upon the partner’s previous behaviors. Here, we used a multinomial processing tree model to distinguish between positive and negative reciprocity and cooperation bias in a sequential Prisoner’s Dilemma game. In Experiments 1 and 2, the facial expressions of the partners were varied to manipulate cooperation bias. In Experiment 3, an extinction instruction was used to manipulate reciprocity. The results confirm that people show a stronger cooperation bias when interacting with smiling compared to angry-looking partners, supporting the notion that a smiling facial expression in comparison to an angry facial expression helps to construe a situation as cooperative rather than competitive. Reciprocity was enhanced for appearance-incongruent behaviors, but only when participants were encouraged to form expectations about the partners’ future behaviors. Negative reciprocity was not stronger than positive reciprocity, regardless of whether expectations were manipulated or not. Experiment 3 suggests that people are able to ignore previous episodes of cheating as well as previous episodes of cooperation if these turn out to be irrelevant for predicting a partner’s future behavior. The results provide important insights into the mechanisms of social cooperation.

## Introduction

Cooperation pervades all aspects of human life. Many human achievements including scientific discoveries would be impossible in the absence of cooperation. Therefore, it is important to understand what facilitates and hinders cooperation between individuals. To illustrate, let us imagine that a scientist is approached by a colleague who wants to collaborate on a joint project. The collaboration can be very successful when both partners contribute equally to the project. However, joining the project is also risky– the scientist might end up doing all of the work while the colleague contributes nothing but still claims credit for himself. What determines whether or not the scientist will agree to cooperate with the colleague? Some aspects of the situation may lead the scientist to construct the situation as a cooperative one. For instance, the scientist will be more likely to engage in cooperation when approached in a friendly manner. However, the scientist’s willingness to cooperate is probably much higher when that colleague has previously spent significant time and effort on joint projects. Likewise, the willingness to cooperate may decrease sharply when the scientist remembers that the colleague is notorious for leaving the hard work to somebody else. This illustrates the reciprocal nature of cooperation: one’s own cooperation is often contingent on the other’s behaviors in previous interactions.

Here, we introduce a simple multinomial processing tree (MPT) model [1, 2] to separate three basic forms of cooperation in a sequential Prisoner’s Dilemma (PD) game [3]: positive reciprocity, negative reciprocity, and cooperation bias. Reciprocity is defined as being dependent on the partner’s cooperative or noncooperative behavior in the past. Positive reciprocity is cooperative behavior that is motivated by a partner’s cooperation in previous encounters. Negative reciprocity is uncooperative behavior that is motivated by a partner’s previous defection. Cooperation bias is defined as cooperative behavior that is independent of the partner’s past behavior. We are going to examine whether these forms of cooperation can be manipulated independently of each other, and test whether there is a basic negative-positive asymmetry in reciprocity.

The example at the beginning of this article illustrates the advantages as well as the risks of cooperation. More can be achieved through cooperation, but for each individual, making a cooperative move is risky because it is associated with costs: in order for the cooperation to be mutually beneficial, each individual has to contribute resources (e.g. time, effort, or money). This creates a conflict between individual and collective interests: each individual may be better off by shying away from the costs of cooperation, but collectively the outcome is better if both cooperated [4]. The PD is a standard paradigm for examining this core social dilemma that underlies many forms of cooperation [4–6]. A typical payoff matrix of the PD is shown in Table 1. The defining characteristic of the PD is that the temptation (T) for unilaterally defection is higher than the reward (R) for mutual cooperation, which in turn is better than the punishment (P) for mutual defection, which is still higher than the sucker’s payoff (S) that a cooperative player gets when cheated (T > R > P > S). This payoff structure ensures that each player individually benefits from defecting (regardless of what the other player does), but if both players follow their self interest, they are collectively worse off than if both cooperated.

**Table 1:** Player 1’s payoff in a typical Prisoner’s Dilemma (PD).

		Player 2	
		“I cooperate”	“I do not cooperate”
**Player 1**	**“I cooperate”**	Reward (R)	Sucker’s Payoff (S)
		10	-10
	**“I do not cooperate”**	Temptation (T)	Punishment (P)
		20	0

The sequential PD game [3] maintains this payoff structure, but introduces a delay between the choices of the first and the second player. After observing the cooperation or defection of the first player, the second player chooses to cooperate or to defect. In the present study, this helps to disambiguate the behavior of the second player because the defection of the second player after a cooperative move of the first player is clearly characterized by a selfish disregard of fairness [3], and can, therefore, be labelled as cheating.

Rationality in terms of maximizing one’s self-interest dictates that both players should choose “defect” in PD games. However, reciprocal strategies can prevail in repeated games. For instance, tit for tat can outcompete egoistic strategies in computer simulations [6]. This reciprocal strategy consists of comparatively simple rules: cooperate if you interact with someone for the first time. Then, just copy what the other person did in the subsequent round. However, even though game-theoretical simulations were very successful in identifying candidate mechanisms for human cooperation [7], they often imply assumptions about the underlying cognitive architecture that require empirical validation [8, 9]. Reciprocal strategies, for instance, rely heavily on memory. Only when the behavior of an interaction partner is reliably remembered, cooperation and defection can be effectively reciprocated [9–11]. Empirical studies have shown that memory for cooperation and defection is far from perfect, leading to memory errors that can decrease the discriminatory power of reciprocity [9]. Furthermore, it has been noted that even individuals with a propensity for cooperation do not indiscriminately begin with a cooperative move when interacting with strangers, but instead carefully adapt their strategies to their understanding of the specific situation [12]. Therefore, the computer-simulation approach has to be complemented by the empirical examination of the cooperative strategies that people actually use.

A recurring issue in the study of cooperation is whether the ability to identify and deal with defection is particularly significant [10, 13–17]. In principle, reciprocal strategies can be maintained by attending to and remembering either cheaters or cooperative individuals [18–20]. However, because cooperation is often considered the default strategy, many evolutionary theories imply that it is particularly important to discriminate against cheaters [13, 16]. Specifically, social contract theory [21] starts from the assumption that most people have a strong bias for cooperation. Given that unconditional cooperation is not an evolutionary stable strategy, this propensity to cooperate has to be balanced out by an evolved cheater-detection mechanism that helps them to immediately and reliably reciprocate a partner’s cheating. The theory was originally developed to account for peculiarities of human reasoning, but it can be generalized to predict that people (a) reciprocate noncooperative behaviors more reliably than cooperative behaviors [17, 22], and (b) remember a partner’s cheating better than a partner’s cooperation [10, 15, 23].

In the domain of memory, many studies have examined how reliably cooperation and cheating is remembered [11]. Although earlier studies supported the cheater-detection hypothesis [10, 14, 15, 23], recent studies provide support for a more flexible mechanism [19, 24–27]. Bell et al. [26] examined their participants’ memory for the behaviors of partners in a sequential PD game. When the partners looked trustworthy, participants were more likely to guess that an unknown partner was a cooperator, but memory was better for their partners’ cheating than for their partners’ cooperation. However, when the partners looked untrustworthy, the opposite pattern emerged: Participants were more likely to guess than an unknown partner was a cheater, but memory was better for their partners’ cooperation than for their partners’ cheating (see also [28, 29]). Bell et al. [26] argued that this expectancy-violation mechanism is better suited to facilitate social cooperation than a cheater-detection module. Specifically, situational and facial cues may elicit strong tendencies to trust or to distrust other persons. Enhanced memory for expectancy-violating social information may play an important role in correcting these tendencies in situations where such cues are misleading.

Obviously, this functional interpretation implies that better memory translates directly into increased reciprocity of expectancy-violating behaviors. However, memory biases and cooperative tendencies in the sequential PD game may dissociate for a number of reasons. For instance, people may have equal memory for cooperation and cheating, but they may be more influenced by memory of cheating when making PD decisions because they think that the negative information is more diagnostic. Furthermore, people may have more confidence in memory for expectancy-confirming events, which could lead them to ignore their good memory for expectancy-violating events. Given these considerations, direct empirical evidence is needed to understand how people integrate expectancy-violating information into their PD decisions.

In the exposure phase of the present Experiment 1, participants played a sequential PD game with partners whose facial expressions were manipulated, as in Experiment 2 of Bell et al. [26]. Contrary to that previous study, the memory test was replaced by a test-phase sequential PD game. Different from the participants in the previous study [26]—who had to identify the partners as cooperators, cheaters and new partners—, the participants in the present study were simply required to decide whether they wanted to cooperate with the partners or not. This experiment serves to test whether the enhanced memory for appearance-incongruent behaviors [observed by 26] translates into increased reciprocation of appearance-incongruent cooperation and cheating. A second novel contribution of the present work is that we introduce an MPT model that serves to separately measure reciprocity and cooperation bias. This model is described in the next section.

### Measuring reciprocity and cooperation bias

MPT models represent a well-established class of measurement models for categorical data in Cognitive Psychology [1, 2, 30]. They specify how observed response frequencies in a set of response categories originate from combinations of cognitive operations that can be illustrated in a tree-like structure [31]. MPT models allow measuring the probabilities with which these cognitive processes occur [2]. This is achieved by minimizing the log-likelihood ratio goodness-of-fit statistic *G*^2^, following the expectation-maximization algorithm proposed by Hu and Batchelder [32]. Model identifiability is given when a set of response frequencies map to a unique set of parameter estimates [2]. A big advantage of MPT models is that statistical tests can be performed directly at the level of the model parameters. Over the past decades, this method has been successfully applied to measure cognitive states, processes, and decisions in areas such as memory [33–37] and decision making [38–40], and to test statistical hypotheses about them. Several computer programs are available for parameter estimation and goodness-of-fit tests [31, 41, 42].

**Fig 1:**
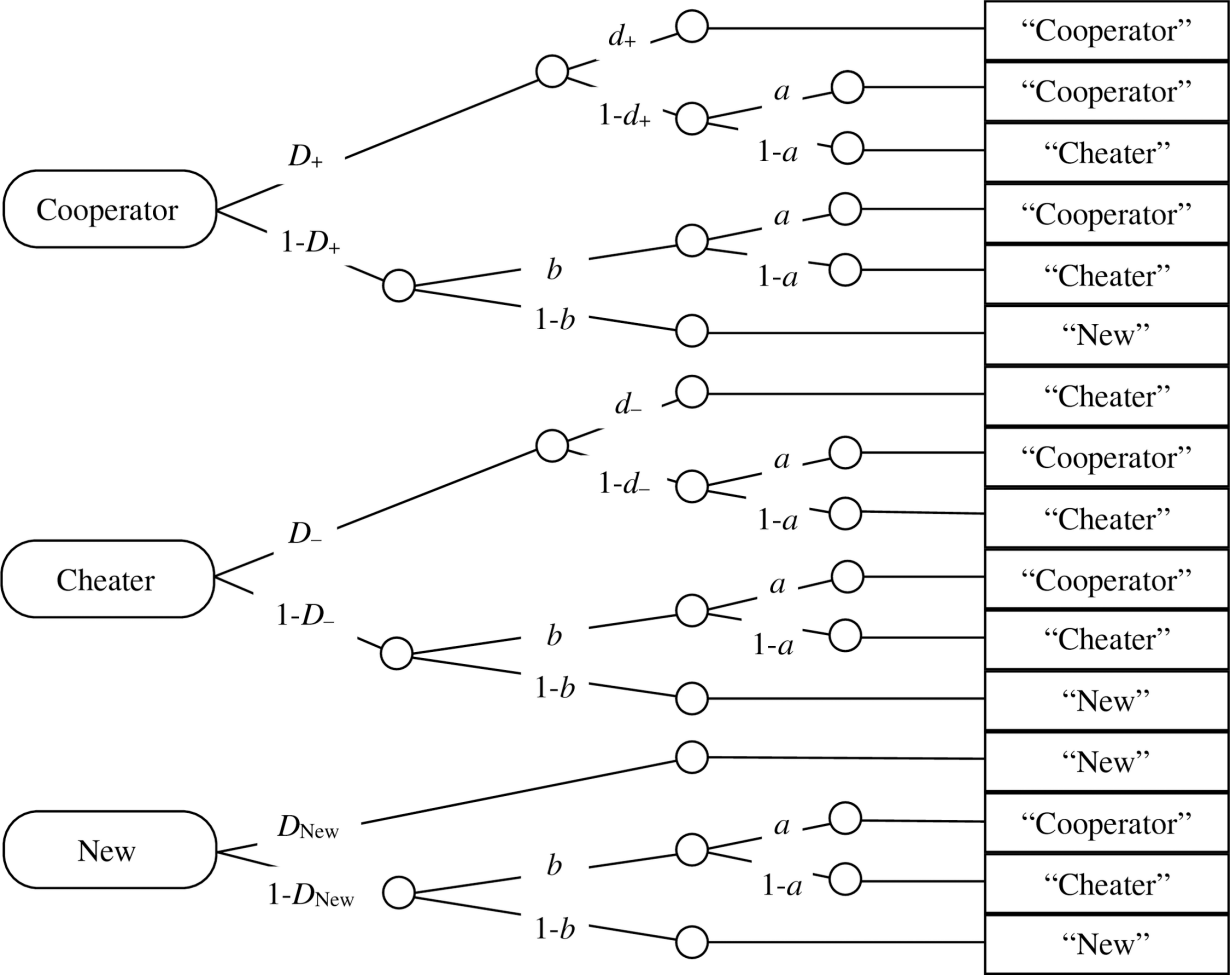
The source monitoring model of Bayen, Murnane, and Erdfelder (1996). Rounded rectangles represent the type of faces presented in the test (cooperators, cheaters, and new faces). Rectangles on the right represent the participant’s decisions in the memory test (“Cooperator”, “Cheater”, “New”). The letters along the links represent the probability of different cognitive processes (*D*_+_: recognizing the face of a cooperator as old; *d*_+_: remembering that the face was associated with cooperation; *D*_–_: recognizing the face of a cheater as old; *d*_–_: remembering that the face was associated with defection; *D*_New_: recognizing a new face as new, *b*: guessing that an unrecognized face was old; *a*: bias towards guessing that a face was associated with cooperation rather than defection).

To analyze the present data, we used an MPT model to distinguish between two basic forms of cooperation: reciprocity and cooperation bias. Reciprocity mirrors a partner’s previous cooperation or cheating while cooperation bias is defined as being independent of the partner’s previous behavior. This is parallel to how the source monitoring model used in our previous work [10, 24, 26, 43] distinguishes memory from guessing. According to the widely used source monitoring model shown in Fig 1 [44, 45], the classification of a face as belonging to a “cooperator” or a “cheater” is due to a guessing bias if it is independent of whether the face was actually paired with cooperation or cheating in the exposure phase. Therefore, the same guessing parameter *a* is used for cooperator faces, cheater faces, and new faces. Finding that all of those faces are uniformly classified as “cooperators” rather than as “cheaters” would be evidence of a guessing bias rather than memory. In contrast, finding that cooperator faces are more likely to be classified as “cooperators” than other faces would represent evidence of memory. Accordingly, the model includes two source memory parameters *d*_+_ and *d*_−_that represent the probabilities of remembering that faces were associated with cooperation or cheating, respectively, which leads to correct classifications of these faces as “cooperators” and “cheaters”, above of what would be expected due to guessing alone.

**Fig 2:**
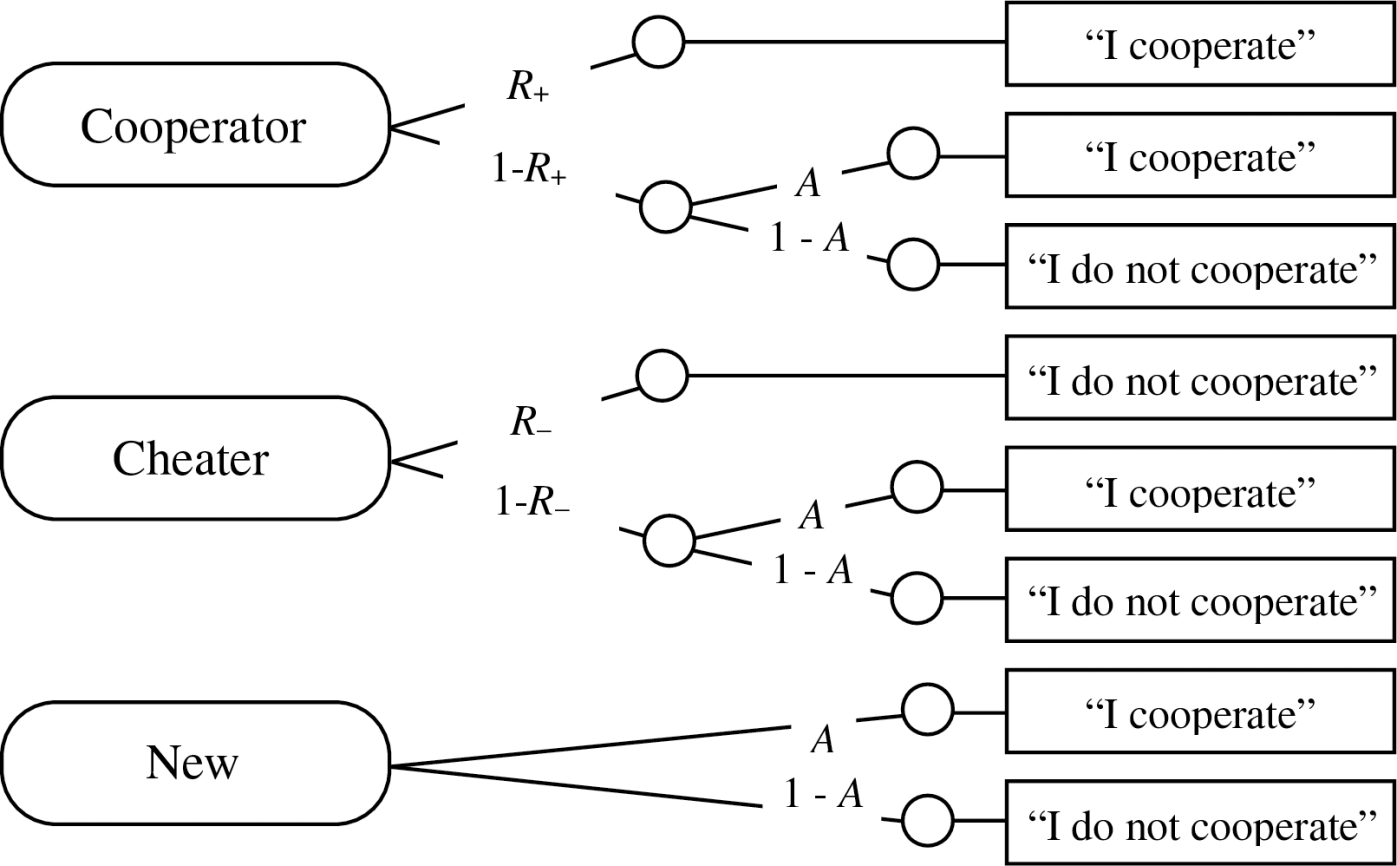
The reciprocity model used in the present experiments. Rounded rectangles on the left side represent the types of partners presented in the test (cooperators, cheaters, and new faces). Rectangles on the right represent the participants’ decisions (“I cooperate”, or “I do not cooperate”). The letters along the links represent the probabilities of different strategies (*R*_+_: positive reciprocity; *R*_–_: negative reciprocity; *A*: cooperation bias).

When the source memory test is replaced by a test-phase sequential PD game, the same method can be applied to distinguish between reciprocity and cooperation bias. The categorical nature of the data obtained in social dilemma games lends itself to analyses with MPT models [40]. In the test phase of the present study, participants were simply asked to decide whether they wanted to cooperate with the partner or not. According to the model shown in Fig 2, it is possible that the participants’ decisions are determined by reciprocity, in which case they would mirror the partners’ exposure-phase decisions. There are two types of reciprocity that are distinguished by the model. Participants may show evidence of positive reciprocity (*R*_+_) by reciprocating the cooperation of the cooperative partners, and they may show evidence of negative reciprocity (*R*_–_) by reciprocating the noncooperation of the cheaters. Alternatively, decisions to cooperate may be independent of the partners’ previous behaviors, which would be evidence of a cooperation bias (*A*). A bias of *A* > .50 would reflect a propensity for cooperation while a bias of *A* < .50 would reflect a propensity for noncooperation.

Note that the model is structurally similar to the source monitoring component of the well-established source monitoring model [44] shown in Fig 1, with which it shares the property of being identifiable. However, this similarity refers only to the structure of the model and not to the interpretation of the parameters, which necessarily differs between the two paradigms. The item recognition and source memory parameters estimated by the source monitoring model depicted in Fig 1 reflect retrospective memory components. In the test phase of the present experiments, participants were simply asked whether they wanted to cooperate with the partner or not. No explicit reference was made to the previous episode of cooperation or cheating. Although reciprocity is impossible without memory and reflects the influence of the past experiences with the partners, participants have to make prospective judgments about whether or not the partners deserve their trust, and they may choose to ignore their memory for their partners’ previous behaviors. The reciprocity parameters estimated by the model depicted in Fig 2 reflect this prospective trust-based process.

To illustrate the utility of the model, consider, for instance, occasions at which participants decide to cooperate with cooperative partners. These decisions to cooperate may be either due to positive reciprocity (with probability *R*_+_) or due to a lack of reciprocity combined with a bias for cooperation [with probability (1 – *R*_+_)·*A*]. Thus, the probability with which a person cooperates with a cooperative partner represents a mixture of these different processes. By using the MPT model, it is possible to decompose the participants’ decisions for or against cooperation into the processes involved, and to separately estimate the probabilities associated with positive and negative reciprocity and cooperation bias. What is more, statistical tests can be performed directly at the level of these model parameters. For instance, the hypothesis that negative reciprocity is stronger than positive reciprocity implies that the model parameter representing negative reciprocity *R*_−_is larger than the model parameter for positive reciprocity *R*_+_. It can be tested by imposing onto the model depicted in Fig 2 the restriction that *R*_−_ = *R*_+_. This restricted model can then be fitted to the data. If the fit of the restricted model is significantly worse than the fit of the model without this restriction (and if, at the level of the estimates, *R*_−_> *R*_+_), we would have to reject the assumption *R*_−_ = *R*_+_, and conclude that negative reciprocity is stronger than positive reciprocity. If, in contrast, the assumption *R*_−_ = *R*_+_ does not significantly decrease the model fit, we would have to conclude that the data do not allow us to reject the assumption *R*_−_ = *R*_+_, and conclude that the hypothesis that negative reciprocity is stronger than positive reciprocity is not supported by the results.

### Hypotheses

A common requirement for the acceptance of MPT models is that the models’ parameters respond to experimental manipulations of the processes reflected in these parameters [44]. In the present case, the model’s parameters *R*_+_, *R*_–_, and *A*, should be sensitive to manipulations of reciprocity and cooperation bias, respectively. Specifically, we predicted that the participants’ cooperation bias *A* should be higher when interacting with smiling partners than when interacting with angry-looking partners in Experiments 1 and 2, based on evidence showing that smiling, in comparison to an angry facial expression, increases cooperation among strangers in one-shot interactions [46], and leads people to interpret ambiguous situations as cooperative rather than competitive [47]. Experiment 3 completes the model validation by using an extinction instruction to directly manipulate the reciprocity parameters *R*_+_ and *R*_–_.

Furthermore, two conflicting hypotheses were tested by Experiments 1 and 2. Based on our previous studies [24, 26, 48], we postulated that appearance-incongruent behaviors should be reciprocated with a higher probability than appearance-congruent behaviors because expectancy-violating events attract attention, and are remembered better than expectancy-confirming events. For instance, people should have better memory for the cheating of a smiling person (an expectancy-violating event), and they should use this memory to correct the false trust that is automatically elicited by the smiling face. This line of reasoning leads to the hypothesis that negative reciprocity should be stronger than positive reciprocity (*R*_−_> *R*_+_) when the participants are interacting with smiling partners, but the opposite should be true (*R*_*+*_ > *R*_*–*_) when they are interacting with angry-looking partners. Social contract theory [21], in contrast, predicts that negative reciprocity should always be stronger than positive reciprocity (*R*_−_> *R*_+_), regardless of the facial expressions of the partners. Experiment 3 tests the additional hypothesis that negative reciprocity is more persistent than positive reciprocity when the previous experiences of cooperation or cheating are invalidated by extinction instructions.

## Experiment 1

### Materials and methods

The study was approved by the ethics committee of the faculty of mathematics and natural sciences at Heinrich Heine University Düsseldorf. Written informed consent was obtained from the participants.

The participants were 120 students at Heinrich Heine University Düsseldorf (76 of whom were female, mean age = 23 years, *SD* = 4) who were recruited on campus. All experiments were conducted in the lab using iMac computers. The participants knew that they played for real money, and that their decisions would influence their payoffs.

Color photographs (768 × 576 pixels) of 36 male and 36 female frontal faces were taken from the AR face database [49]. Two photographs of each individual face were selected, one showing a smiling facial expression and the other showing an angry facial expression.

Participants played two games with the same partners. The first game served to familiarize the participants with their partners, and to give them opportunity to learn about their (cooperative or uncooperative behavior). The second game served to test whether, and how, this experience would affect the participants’ willingness to cooperate with these partners. To avoid confusion, we henceforth refer to the first game as the exposure-phase game, and to the second game as the test-phase game.

**Fig 3:**
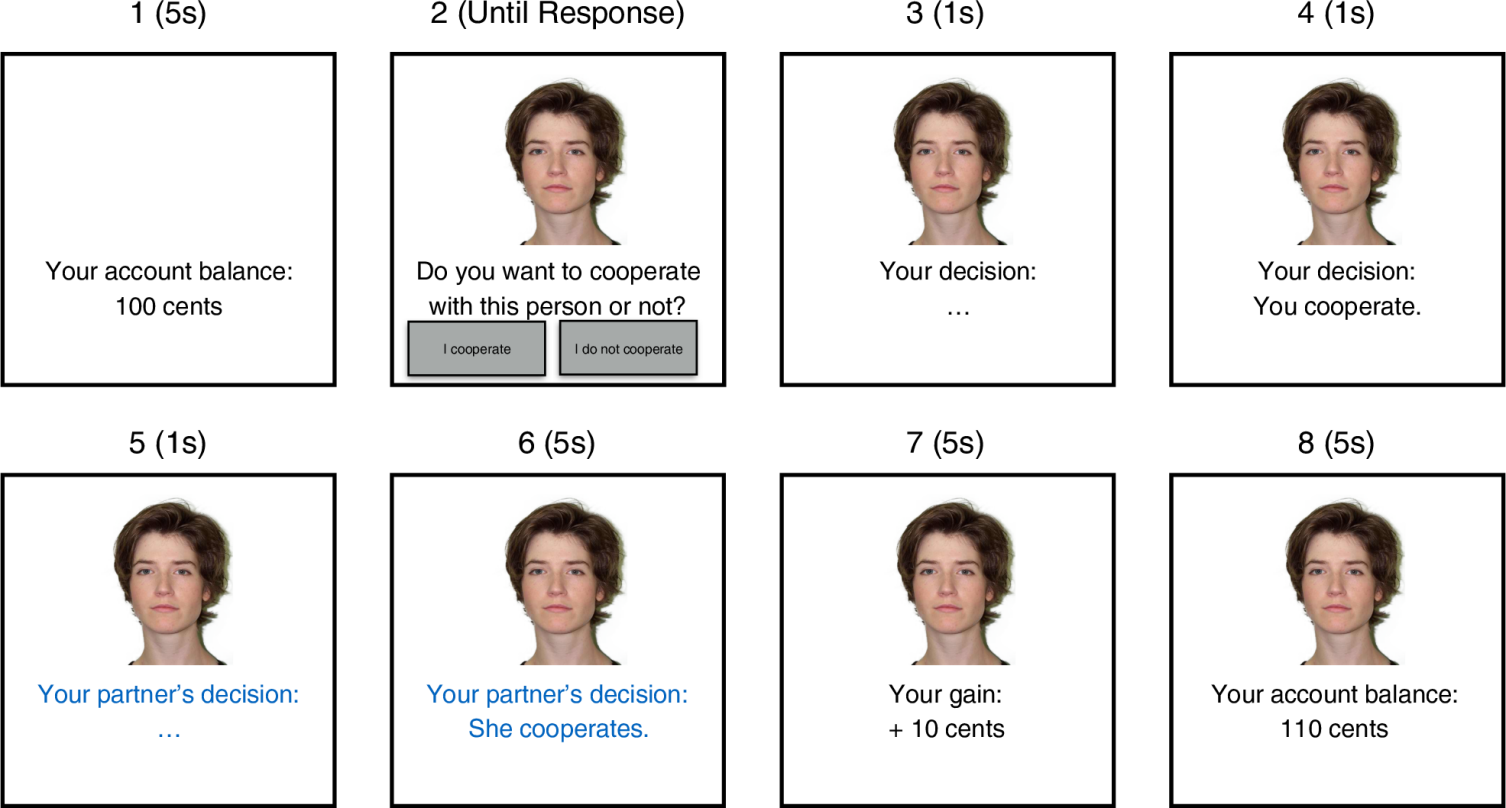
Schematic illustration of a trial of the sequential Prisoner’s Dilemma game. The example face was taken from the Center of Vital Longevity database [67].

The exposure-phase game was similar to the one used in previous studies [24, 43, 50]. A schematic illustration of a single trial is shown in Fig 3. The partner’s face was shown at the center of the screen. The participant was asked “Do you want to cooperate with this person or not?”. There were two buttons, one was labeled “I cooperate” and the other was labeled “I do not cooperate”. As in previous experiments [24, 26, 29, 43], the participant was required to cooperate during the exposure phase (here the “I do not cooperate” option was greyed out) to ensure that the participant received feedback about the cooperation or cheating of the partner. Upon clicking the “I cooperate” button, the question was replaced by the text “Your decision: …” in black font color, which was completed by the information “You cooperate” 1 s later. The participant knew that the partner would be informed about the decision. There were two types of partners. A cooperative partner chose to cooperate. A cheater, in contrast, did not cooperate. After 1 s, the feedback about the participant’s decision was replaced by “Your partner’s decision: …” in blue font color, which was completed by an information about the partner’s decision (“He/She cooperates.” or “He/She does not cooperate.”) 1 s later. The feedback about the partner’s decision was shown for 5 s. Then the participant’s gain or loss was presented in black font color (“Your gain: +10 cents” or “Your loss: –10 cents”) for 5 s (the computation of gains and losses depending on the partner’s cooperation and cheating is described in the subsequent paragraph). The participant’s updated account balance was shown for 5 s. Then the next trial started, and the face of the next partner was shown. In the exposure phase, each participant saw 24 smiling faces and 24 angry faces. Half of the smiling and half of the angry partners cooperated, and the other half refused to cooperate. The faces were randomly drawn from the set of 72 faces, and randomly assigned to conditions. Partner gender was counterbalanced between conditions.

The participant knew that the partner could choose to cooperate or to cheat. A cooperative partner invested as much as the participant (30 cents), which resulted in a net investment of 60 cents. To this net investment, a bonus was added, which was always 1/3 of the net investment (20 cents). The total sum (80 cents) was split up between the participant and the partner, and each of them received half of the total sum (40 cents), regardless of the individual contribution to the game. Thus, mutual cooperation resulted in a gain of 10 cents for each partner. However, a cheating partner did not cooperate and contributed nothing. Therefore, the cheater had no costs, but still received half of the total sum (40 cents), which resulted in a large gain (20 cents). The participant, in contrast, lost money (–10 cents) because the return (20 cents) was too low to compensate the investment (30 cents), due to the absence of the cheater’s contribution.

After the exposure phase, participants received the instructions for the test phase. They were informed that they again played for real money. The main difference to the exposure phase was that participants were now allowed to choose both the “I cooperate” and the “I do not cooperate” option. If they chose the latter option, they would invest no money, and would neither gain nor loose any money. The participants did not receive any feedback about the outcomes of the interactions because we did not want the participants’ decisions to be biased by test-phase feedback. The participants saw 36 smiling (the 12 cooperators, the 12 cheaters, and 12 new faces) and 36 angry-looking faces (the 12 cooperators, the 12 cheaters, and 12 new faces). The participants knew that their final payoffs were determined by their responses in the test-phase game (i.e., relative to an initial endowment of € 1, they lost money when cooperating with defecting partners, and gained money through mutual cooperation).

We used a comparatively large sample of 120 participants to be able to detect even a small effect of *w* = .04 in the comparison of the *A* parameters between conditions (smiling vs. angry) with a sufficient statistical power of 1 – β = .95 [51].

### Results and discussion

To analyze the results of Experiment 1, we needed two sets of the trees depicted in Fig 2, one for interactions with smiling partners and one for interactions with angry-looking partners. The base model fit the data perfectly, *G*^2^ = 0.00. As predicted, the cooperation bias *A* was clearly affected by the partners’ facial expressions, as is evidenced by the fact that restricting parameter *A* to be equal for smiling and angry partners was incompatible with the data, Δ*G*^2^(1) = 41.05, *p* < .01. A smile increased the cooperation bias *A* in comparison to an angry expression (Table 2).

**Table 2:** Estimates of the parameters for cooperation bias (*A*) and positive (*R*_*+*_) and negative (*R*_*–*_) reciprocity as a function of the facial expressions of the partners (smiling vs. angry) with bootstrapped standard errors in Experiment 1.

Facial Expression	*A*	(*SE*)	*R*_+_	(*SE*)	*R*_–_	(*SE*)
**Smiling**	.51	0.01	.29	0.03	.20	0.03
**Angry**	.40	0.01	.30	0.03	.25	0.04

Facial expression had no effect on reciprocity. Restricting parameters *R*_+_ and *R*_−_to be equal for smiling and angry facial expressions fit the data well, Δ*G*^2^(2) = 1.24, *p* = .54. In contrast to the idea that cheating receives special attention [21], negative reciprocity was not stronger than positive reciprocity. If anything, there was a tendency in the opposite direction: Positive reciprocity was somewhat stronger than negative reciprocity. However, this difference was not significant. The restriction that the *R*_+_ parameters did not differ from the *R*_−_parameters was compatible with the data, Δ*G*^2^(2) = 4.05, *p* = .13.

The results of Experiment 1 confirm that parameter *A* responds to the partners’ facial expressions as expected. The cooperation bias was higher when participants interacted with smiling partners than when they interacted with angry-looking partners. This confirms the view that the cooperation bias depends on cue-based judgments about the likelihood of the partner’s cooperation. Specifically, a smiling facial expression helps people to construe a situation as cooperative [47] and increases cooperation [46], while an angry facial expression leads people to construe the situation as competitive [47] and decreases cooperation [26].

Reciprocal cooperation, in contrast, was not affected by the facial expression of the partner. This finding is welcome with respect to the model validation because it shows that the model’s parameter *A* can be manipulated without affecting the *R* parameters. However, at first glance, this finding is surprising when considering previous reports of an incongruity effect on memory. In some previous experiments, it has been shown that people have enhanced memory for behavior that is incongruent with expectations [19, 25, 28, 52]. For example, people had better memory for cheaters than for cooperators when the partners were smiling, and this memory advantage was descriptively reversed when the partners had an angry facial expression [26]. Given that memory (and, in particular, memory for the association between the partners’ faces and their cooperation or cheating in the exposure-phase game) can be considered a necessary prerequisite for reciprocal behavior, it could be postulated that reciprocity should be stronger in the incongruent conditions. Such a pattern of results has been obtained by Suzuki et al. [22], who found that being informed that a trustworthy-looking person was “bad” and that an untrustworthy-looking person was “good” led to comparatively strong changes in the reciprocal component of cooperation.

It is plausible that the effect of expectancy violations is stronger when the way in which the partners are encountered encourages the forming of expectations first. This was the case in the study of Suzuki et al. [22], in which participants first decided whether or not to trust a partner in a lending game before being informed about their partner’s “good” or “bad” character. To avoid the problem that untrusting participants cannot experience cooperation or cheating, Suzuki et al. imposed a constraint on the number of times the “I do not trust” option could be selected. Nevertheless, this procedure probably stimulated participants to form expectations about their interaction partners’ future behaviors. In other studies on memory for cooperators and cheaters (e.g., [26, 43]), participants were required to form expectations about their partners because they were required to decide whether they wanted to invest 15 or 30 cents in the exposure-phase game before the partners’ decisions were revealed. Investing the larger amount of money was only profitable when the partners were trustworthy. Therefore, the participants had to decide whether they trusted their partners enough to invest the larger amount of money. In the present Experiment 1, in contrast, participants were required to always cooperate in the exposure-phase game; the “I do not cooperate” option was only available in the test-phase game. In consequence, they did not have to form expectations about whether or not to trust the partners in the exposure phase.

Interestingly, a previous study suggests that the memory advantage for incongruent information may disappear when the way in which the partners are encountered during the exposure phase does not directly encourage the forming of expectations about the partners’ behaviors. Mattarozzi et al. [53] found no evidence of enhanced memory for appearance-incongruent behaviors, and speculated that the absence of an incongruity effect in their study was due to the fact that they did not require participants to evaluate the trustworthiness of the persons (e.g., by deciding whether or not to trust these persons in a social dilemma game) before the incongruent behavioral information was displayed. According to this view, expectancy violation results in a memory benefit only when participants are explicitly required to form expectations about the partners’ trustworthiness.

## Experiment 2

Experiment 2 serves to test whether an incongruity effect on reciprocal cooperation emerges when the way in which the partners are encountered during the exposure phase encourages the forming of expectations about the partners’ trustworthiness. As in previous experiments [26, 43], participants were required to decide how much money they wanted to invest into the exposure-phase game. Investing a higher amount of money led to a larger benefit from mutual cooperation, but also to a greater loss when the partner refused to cooperate. Investing a small amount of money led to a smaller benefit from mutual cooperation, but also to a smaller loss when the partner turned out to be a cheater. Therefore, the investment decision depended crucially on the amount of trust placed in the partner. If the expectancy violation effect emerges when the participants are encouraged to form expectations about their partners’ behaviors, unexpected behaviors should be reciprocated with a higher probability than expected behaviors in Experiment 2. In addition, Experiment 2 serves as a replication of the effect of facial appearance on the cooperation bias *A*.

### Materials and methods

Participants were 144 students at Heinrich Heine University Düsseldorf (77 of whom were female, mean age = 24 years, *SD* = 5) who were recruited on campus.

Stimuli and procedure were identical to those of Experiment 1 with the exception that participants were required to decide how much money they wanted to invest in the game. This procedure is identical to that of previous experiments in which evidence of an incongruity effect on source memory has been obtained [26]. In the exposure phase, participants were asked “How much do you want to invest?” and were required to decide whether they wanted to invest 30 cents or 15 cents. Investing the higher amount of money (30 cents) meant a higher reward in case of mutual cooperation (a reward of 10 cents), but also a higher risk (a loss of 10 cents) when the partner decided to cheat. Investing the lower amount of money (15 cents) meant less reward for mutual cooperation (a reward of 5 cents), but also a lower cost (a loss of 5 cents) of being cheated. The test phase was identical to that used in Experiment 1: participants had to choose whether they wanted to cooperate with the partners or not, and no direct feedback was provided.

Given that Experiment 2 served to test whether a different pattern of results in the *R* parameters could be obtained with a somewhat different paradigm, we aimed at recruiting about the same number of participants as in Experiment 1. The experiment had a statistical power of 1 – β > .99 to replicate the effect of facial expression on the *A* parameter.

### Results and discussion

We used the same base model as in Experiment 1. This model fit the data perfectly, *G*^2^ = 0.00. As in Experiment 1, and consistent with our expectations, cooperation bias (reflected in parameter *A*) was significantly higher for interactions with smiling partners than for interactions with angry-looking partners, Δ*G*^2^(1) = 91.17, *p* < .01 (Table 3).

**Table 3:** Estimates of the parameters for cooperation bias (*A*) and positive (*R*_*+*_) and negative reciprocity (*R*_*–*_) as a function of the facial expressions of the partners (smiling vs. angry) with bootstrapped standard errors in Experiment 2.

Facial Expression	*A*	(*SE*)	*R*_+_	(*SE*)	*R*_–_	(*SE*)
**Smiling**	.42	0.01	.15	0.03	.20	0.04
**Angry**	.27	0.01	.23	0.02	.10	0.05

Descriptively, reciprocity of appearance-incongruent behaviors was higher than that of appearance-congruent behaviors (i.e., the cheating of smiling partners and the cooperation of angry-looking partners were more likely to be reciprocated than the cooperation of smiling partners and the cheating of angry-looking partners). To formally test the incongruity hypothesis, we used a base model with one parameter *R* representing reciprocity in the incongruent conditions and one parameter *R* representing reciprocity in the congruent conditions, which fit the data well, *G*^2^(2) = 0.94, *p* = .63. Next, we tested whether reciprocity was higher in the incongruent conditions compared to the congruent conditions, and this was the case, Δ*G*^2^(1) = 6.40, *p* = .01.

Experiment 2 successfully replicated the finding of Experiment 1 that the facial expressions of the partners affected cooperation bias. As in Experiment 1, parameter *A* was higher when the participants interacted with smiling partners than when they interacted with angry-looking partners. Thus, parameter *A* captures differences in cooperation bias.

In addition, an incongruity effect was obtained in the *R* parameters. Behaviors that violated participants’ expectations were more likely to be reciprocated. The simplest explanation of the difference between Experiments 1 and 2 is that an expectancy violation effect can only be observed when the task encourages participants to build up expectations about the partners’ future behaviors, consistent with previous findings showing that the processing of expectancy-violating feedback depends on the participants’ active involvement in the game [54].

The results of Experiment 2 also provide further evidence that it makes sense to distinguish between different forms of cooperation because the facial expressions of the partners had differential effects on cooperation bias and reciprocity. This is consistent with the previous suggestion that people base cooperative decisions on immediately available situational and facial cues, but may also use more effortful reciprocal strategies to correct these appearance-based biases when the situational and facial cues are misleading [11]. The tendency to reciprocate expectancy-incongruent behaviors (e.g., to increase cooperation with untrustworthy-looking individuals who have proven to be cooperative) may therefore complement the expectancy-congruent cooperation bias.

## Experiment 3

So far, the results fit well with the idea that participants flexibly adapt their prioritization of positive and negative information to their expectations [11, 19, 24, 26], but are inconsistent with an inflexible prioritization of negative information. Specifically, Experiment 1 shows that negative reciprocity is not stronger than positive reciprocity when the paradigm does not explicitly encourage the forming of expectations about the partners’ behaviors during the exposure phase. In fact, positive reciprocity was numerically somewhat more pronounced than negative reciprocity. In Experiment 2, participants reciprocated expectancy-violating behavior more than expectancy-congruent behavior, but there was no negative-positive asymmetry in this expectancy violation effect. The reciprocation of expectancy-violating positive behavior was at least as large as (and, numerically, even somewhat more pronounced than) that of expectancy-violating negative behavior. This is inconsistent with the theoretical emphasis on cheater detection [21], and suggests that the reciprocation of friendly acts is as important for establishing and maintaining cooperation as the retaliation of cheating.

However, there is a further assumption about the negative-positive asymmetry in the processing of social information that remains to be tested. Specifically, it is often assumed that negative impressions are sticky in the sense that they are maintained even when the reasons for the negative judgments are invalidated [55, 56]. In line with this idea, Suzuki et al. [22] have proposed that the previous cheating of a partner—in contrast to the previous cooperation of a partner—has more persistent effects on later behavior even when participants know that previous behavior should be ignored. To test this hypothesis, Suzuki et al. instructed their participants in an extinction group that the previously experienced face-behavior pairings had been randomly determined and that they would be reset in the test phase. Suzuki et al. reported that their participants succeeded in ignoring the previous cooperation of a partner when making test-phase trustworthiness judgments following this extinction instruction, but the previous cheating of a partner continued to influence trustworthiness perceptions. From this finding, Suzuki et al. concluded that memory for cheaters is more persistent against extinction than memory for cooperative individuals. Based on this hypothesis, it is to be expected that negative and positive reciprocity are equivalent when no extinction instruction is given, but evidence for an evolutionary prepared cheater advantage should emerge after an extinction instruction because negative reciprocity has a higher persistence against extinction. In Experiment 3, we used the same extinction instruction as Suzuki et al. to test whether negative reciprocity is more persistent against extinction than positive reciprocity.

In addition, the extinction procedure of Suzuki et al. [22] serves to demonstrate that reciprocity (*R*) can be manipulated independently of cooperation bias (*A*). Experiments 1 and 2 have shown that parameter *A* reflects manipulations of cooperation bias and Experiment 2 has shown that parameters *R*_+_ and *R*_−_respond as predicted to expectancy violations. It remains to be shown that the reciprocity parameters *R* can be selectively manipulated (without affecting cooperation bias). Given that the extinction instruction used by Suzuki et al. [22] should seriously undermine the credibility of the information learned during the exposure phase, it can be hypothesized that the *R* parameters should be reduced by this manipulation if they accurately reflect the reciprocal component of cooperation.

### Materials and methods

One data file was lost because it was not correctly saved. The remaining sample consisted of 33 students at Heinrich Heine University Düsseldorf (19 of whom were female, mean age = 24 years, *SD* = 5) who were recruited on campus. Participants were consecutively assigned to one of the two conditions (i.e., the first participant received the extinction instruction, the second participant did not, and so on).

Stimuli and procedure were identical to those of Experiment 1 with the following exceptions. A total of 36 male faces and 36 female faces with neutral facial expressions were taken from the AR face database [49]. In the exposure phase, participants interacted with 12 cooperators and 12 cheaters. For each participant, the faces were randomly selected and assigned to conditions. Partner gender was counterbalanced between conditions. In the test phase, participants saw 36 faces (12 cooperators, 12 cheaters, and 12 new faces). Half of the participants received written extinction instructions after the exposure phase. We replicated the instructions of Suzuki et al. [22] as closely as possible. Participants were informed that the partners’ behaviors in the exposure phase were randomly determined and that the face-behavior mapping was reset for the test phase. Furthermore, it was explicitly stated that the partners’ behaviors in the test phase would be independent of those shown in the exposure phase, and that the previous game should be ignored. Therefore, the participants’ decisions about whether to cooperate or not should be based on the partners’ appearances but not on their previous behaviors. In essence, then, the participants were required to make their decisions as if they would see the partners for the first time.

The power analysis option of multiTree [42] was used to perform an a priori power analysis. Using the results of Experiment 1 as a basis, we assumed that the cooperation bias for neutral faces would correspond to *A* = .50, and that the reciprocity parameters would correspond to *R*_+_ = *R*_−_ = .26 in the control group. Then we assumed that the *A* parameter would be the same, but the *R* parameters would be zero in the extinction group. The α level was set to .05. To obtain a power of at least 1 – β = .95 for the comparison of the *R* parameters between the groups (given the assumptions spelled out above), it was necessary to recruit at least 32 participants.

### Results and discussion

To analyze the results of Experiment 3, we needed two sets of the trees depicted in Fig 2, one for the control group and one for the extinction group. Again, this base model fit the data perfectly, *G*^2^ = 0.00. The parameter estimates are reported in Table 4. The cooperation bias did not differ significantly between groups, Δ*G*^2^(1) = 1.77, *p* = .18, as expected. Furthermore, positive reciprocity did not differ from negative reciprocity in both groups, Δ*G*^2^(2) = 0.33, *p* = .85. The latter restriction was incorporated into a new base model, which fit the data well, *G*^2^(2) = 0.33, *p* = .85. This base model was used to test whether reciprocal cooperation differed between groups, which was the case, Δ*G*^2^(1) = 11.11, *p* < .01. The extinction instructions greatly reduced and almost abolished reciprocity.

**Table 4:** Estimates of the parameters for cooperation bias (*A*) and positive (*R*_*+*_) and negative reciprocity (*R*_*–*_) as a function of group (control vs. extinction) with bootstrapped standard errors in Experiment 3.

Group	*A*	(*SE*)	*R*_+_	(*SE*)	*R*_–_	(*SE*)
**Control**	.49	0.03	.30	0.08	.35	0.08
**Extinction**	.55	0.04	.13	0.11	.07	0.09

As expected, the extinction instructions used in Experiment 3 greatly reduced participants’ willingness to reciprocate cooperation, but had no significant effect on cooperation bias. Given that the extinction instruction explicitly states that the behavior in the exposure phase is invalid, it may seem intuitively obvious that reciprocity should be abolished, and, at first glance, the results could be dismissed as unsurprising. However, they are non-trivial because they offer direct support for three important points.

First, the results demonstrate that reciprocity can be manipulated without affecting cooperation bias. This finding complements the results of Experiment 1, in which the manipulation of facial attributes of the partners selectively affected cooperation bias, but had no effect on reciprocity. Together, these experiments demonstrate that the reciprocity model’s parameters *A*, *R*_*+*_, and *R*_*−*_can be manipulated independently of each other. Furthermore, Experiment 3 demonstrates that the *R* parameters sensitively reflect the to-be-expected changes in reciprocity.

Second, Suzuki et al. [22] have used the same extinction manipulation to provide support for their hypothesis that memory for cheaters is special because negative experiences are stickier than positive experiences (in the sense that they have effects on subsequent behavior even when invalidated). The results show that this is clearly not the case in the present study. If anything, there was a descriptive, but statistically non-significant tendency towards a more pronounced reduction of negative reciprocity after extinction instructions. Numerically, positive reciprocity survived to a greater extend than negative reciprocity even though this difference was not statistically significant. A closer look on the data of Suzuki et al. reveals that their case for more persistent cheater memory is less compelling than one might think. Specifically, resistance of cheater memory against extinction was only found in facial trustworthiness ratings (that continued to be affected by the previous association with cheating after extinction instructions), but it was not found in the participants’ investments in the lending game. None of the three experiments reported by Suzuki et al. yielded statistically significant evidence of a more robust effect of a partner’s previous cheating—in comparison to a partner’s previous cooperation—on the participant’s decision to invest money in the game. Therefore, both Suzuki et al.’s data and the present Experiment 3 are consistent in showing that participants are well able to ignore a previous episode of cheating when they are explicitly instructed that it is not diagnostic of the partner’s future behavior. The evolutionary significance of an enhanced resistance of cheater memory against extinction can be questioned when it has no effect on reciprocity.

The third point may be less obvious than the first two, but is nevertheless worth mentioning. The results of Experiment 3 suggest that reciprocity should not be equated with memory. This is important because several previous studies have equated memory and reciprocity [17, 22]. However, it is implausible that the extinction instructions caused participants to suddenly forget all of the partners’ previous behaviors because people’s capability to voluntarily suppress memories is extremely limited at best [57]. This suggests that participants were well aware—at least in some cases—that a face was previously associated with an episode of cheating, but chose to ignore it because they knew that it was not predictive of the partner’s future behavior. This finding illustrates that reciprocity is not only retrospectively determined. When people think that their partners’ past behaviors are not informative about their future behaviors, reciprocity will decline. This confirms our view of reciprocity as being both retrospectively and prospectively determined. Reciprocation is often conceptualized as a purely retrospectively motivated behavior, but it may be partly determined by prospective concerns about the likelihood of future cooperation.

## General discussion

To analyze the present data, we have introduced a new MPT model that is able to separately measure reciprocity and cooperation bias. The model was deliberately designed to make only minimal assumptions about cooperation. For instance, we did not a priori assume that negative reciprocity is stronger than positive reciprocity although such an assumption can be empirically tested using the model. Instead, the model only assumes that two basic types of cooperation should be distinguished, one type (reciprocal cooperation) that depends on the partner’s previous behavior and another type (cooperation bias) that is independent of the partner’s previous behavior in the game. A common requirement for the acceptance of an MPT model is that its parameters are sensitive to manipulations of the latent processes which the model is supposed to measure. The present experiments show that the parameters *A*, *R*_+_, and *R*_−_do indeed respond predictably to experimental manipulations of cooperation bias and reciprocity, respectively.

In Experiment 1, the facial expressions of the partners were varied to manipulate cooperation bias. Smiling in comparison to angry expressions of the partners increased cooperation bias while reciprocity was not affected. This confirms the assumption that facial cues can guide the construal of social situations. Specifically, smiling and angry facial expressions can lead participants to interpret situations as cooperative or competitive, respectively [47]. Experiment 2 successfully replicated this effect and provided evidence of the enhanced reciprocation of expectancy-violating behaviors. In Experiment 3, we provided a validation of the reciprocity parameters by manipulating them independently of the cooperation bias. Informing participants that the face-behavior pairings in the exposure phase were invalid led to a decrease of reciprocity, but did not affect cooperation bias. This supports our hypothesis that reciprocity is not only determined by memory for the behavior of the partners, but instead represents a prospective judgment about whether a person can be trusted based on his or her past behavior. When the participants’ confidence in the validity of their memory was undermined, reciprocity was abolished. Overall, the results confirm that the model parameter *A* reflects cooperation bias and that model parameters *R*_+_, and *R*_−_reflect reciprocity. Both types of parameters can be manipulated independently of each other. In summary, the results suggest that the model provides a useful tool for distinguishing between cooperation bias and reciprocity.

In addition, the present experiments served to test the hypothesis of social contract theory [21] that negative reciprocity is stronger than positive reciprocity. This hypothesis was generally disconfirmed by the results: In Experiment 1, there was a nonsignificant tendency for participants to reciprocate cooperation to a greater extent than cheating. In Experiment 2, participants generally tended to reciprocate behavior that violated appearance-based expectations to a greater degree than behavior that was in line with appearance-based expectations. This finding fits to previous reports showing that expectancy-violating behavior is better remembered than expectancy-congruent behavior [19, 24–28, 48]. Importantly, there was no negative-positive asymmetry in this expectancy-violation effect. Participants did not reciprocate unexpected cheating to a larger degree than unexpected cooperation. In Experiment 3, participants were well able to ignore previous episodes of cheating when these turned out to be irrelevant, which is inconsistent with the idea that negative experiences are stickier than positive experiences when invalidated by an extinction instruction [22]. Taken together, the findings are inconsistent with the idea that negative reciprocity is always stronger than positive reciprocity. Instead, people seem to adapt quickly to the relevance of cheating relative to cooperation in a given situation, consistent with the concept of a more flexible use of information in social exchange [11, 19, 43]. This seems to demonstrate that positive reciprocity is at least as important as negative reciprocity. People are generally very concerned about reciprocating others’ favors [58] and building reputations [59]. Finding trustworthy cooperation partners and friends is an important task, just as important as detecting cheaters. At a more general level, the present results are consistent with the idea that cognitive processing is characterized by a prioritization of stimuli with high motivational-behavioral relevance, regardless of their valence [60–63].

At first glance, this view seems to be difficult to reconcile with the abundant evidence in support of a negativity bias [64]. However, even Baumeister et al. [64], who set out to “review evidence pertaining to the general hypothesis that bad is stronger than good” (p. 323) admitted that there are important exceptions from the rule. Specifically, they found that evidence for a negativity bias in memory is “limited” because memory is determined by conflicting goals (such as maintaining a positive mood and remaining alert to dangers). Given that reciprocity crucially depends on memory, it is subject to the same ambiguity. In our own research on cheater memory, we initially found across several studies [10, 23, 65] that memory for negative behavior was enhanced in comparison to memory for the positive behavior, but later studies [24, 26, 29, 43] showed a more mixed picture. However, it seems possible to integrate these seemingly diverging findings into an expectancy-violation account according to which cheating is well remembered not because the human mind comprises an evolved cheater detection module, but because cheating violates expectations about how people should be responding in certain situations [66]. Given that reciprocity depends crucially on memory—and the present research suggests that reciprocity may indeed mirror memory in most situations—, it may be subject to the same rules. This view implies that negative reciprocity is not always stronger than positive reciprocity, but a negativity bias may emerge when strong positive expectations are violated.

## Supporting information

S1 Raw Data Excel sheet containing raw data and summed response frequencies for Experiments 1, 2 and 3. (XLSX)
